# Too Worried to Judge: On the Role of Perceived Severity in Medical Decision-Making

**DOI:** 10.3389/fpsyg.2018.01906

**Published:** 2018-10-09

**Authors:** Àngels Colomé, Javier Rodríguez-Ferreiro, Elisabet Tubau

**Affiliations:** ^1^Section of Cognitive Processes, Department of Cognition, Development and Educational Psychology, Faculty of Psychology, University of Barcelona, Barcelona, Spain; ^2^Institute of Neurosciences, University of Barcelona, Barcelona, Spain

**Keywords:** medical decision, severity perception, probability judgment, affect, numerical format, numeracy

## Abstract

Ideally, decisions regarding one’s health should be made after assessing the objective probabilities of relevant outcomes. Nevertheless, previous beliefs and emotional reactions also have a role in decision-making. Furthermore, the comprehension of probabilities is commonly affected by the presentation format, and by numeracy. This study aimed to assess the extent to which the influence of these factors might vary between different medical conditions. A sample of university students were presented with two health scenarios containing statistical information on the prevalence of breast cancer and hypertension either through icon arrays (*N* = 71) or natural frequencies (*N* = 72). They also received information regarding a preventive measure (mammogram/low-sodium diet) and the likelihood of a positive mammogram or a rich-sodium diet either when suffering or not suffering from the disease. Before seeing the data, participants rated the severity of the disease and the inconvenience of the preventive measure. After reading the health scenario, participants had to rate its difficulty, and how worrisome it was. They had also to rate the prior probability of suffering from this medical condition, and the posterior probability of it, provided a positive mammogram or a rich-sodium diet. Finally, they rated the extent to which they would recommend the preventive measures. All the rates used the same 1 (little)-8 (a great deal) scale. Participants’ numeracy was also assessed. The scenarios differed significantly in perceived severity and worry, with the cancer scenario obtaining higher scores. Importantly, regression analyses showed that the recommendations in the two health scenarios depended on different variables. A model taking into consideration severity and worry rates best explained decisions in the cancer scenario; in contrast, in the hypertension scenario the model that best explained the recommendations comprised both the posterior probability estimate and the severity rate. Neither numeracy nor presentation format affected recommendation but both affected difficulty, worrying and probability rates. We conclude that previous perceptions of the severity of a health condition modulate the use of probabilistic information for decision-making. The roles of presentation format and numeracy in enabling patients to understand statistical information are also discussed.

## Introduction

Passing from a doctor-centered to a patient-centered model of health has led in the last decades to an increase in the interest devoted to informed consent and how to ensure that decisions are indeed knowledgeable. Informed consent should be provided after the patient has understood the purpose, benefits and potential risks of the alternatives proposed. Risks are often conceptualized as a combined function of the probability of a loss and its consequences (Lipkus, 2007). Hence, in health contexts, risk assessment will depend on its probability but also on how severe this risk is considered to be (Haase et al., 2013). Although the probability and the subjective value of the outcome are usually assumed to be independent constructs, Harris et al. (2009) showed that this was not always the case. They found a main effect of probability but, interestingly, estimation at each probability level was higher when the consequences of participants’ decisions were more severe. Harris et al. attributed their effect to the fact that, in case of severe consequences, the costs associated with underestimating probability are high; individuals, therefore, inflate their estimations of the probability of occurrence as a preventive measure. However, this would happen only when participants can make a decision based on these probabilities.

Importantly, Harris et al. (2009) suggested that the effects of outcome severity would be larger under conditions of emotional involvement. If this was the case, understanding how patients make their medical decisions might require an assessment of their comprehension of the objective information conveyed, but also a consideration of how they interpret it on the basis of their background (e.g., their previous perceptions of the disease and remediation proposed or their attitudes toward them) as well as their affect with regard to it.

Affect has been defined as the “specific quality of “goodness” or “badness” (a) experienced as a feeling state and (b) demarcating a positive or negative quality of a stimulus” (p. 312, Slovic et al., 2004). Finucane et al. (2000) considered that people may base their judgments of an item not only on what they thought about it, but also on how they felt about it, and coined the term “affect heuristic” to name this phenomenon. Loewenstein et al. (2001) talked for the first time of the importance of anticipatory emotions, i.e., immediate visceral reactions to risk and uncertainty such as worry or anxiety, and proposed the “risk-as-feelings hypothesis”. According to these authors, apparently erratic decisions might be due to the fact that people’s emotional reactions to risk respond to factors other than the cognitive evaluation of risks, and are largely insensitive to differences in probability. Finally, other studies such as Pachur et al. (2014) have shown that individuals behave differently in affect-rich (e.g., concerning the side effects of a drug) and affect-poor (monetary) contexts which are otherwise equivalent. Pachur et al. (2014) concluded that affect acted as a “spotlight”, focusing people’s attention on outcomes and leading them to neglect statistical information.

Our aim in this study was to investigate whether previous beliefs and affects related to the severity of a given medical condition and a possible preventive measure might influence the extent to which participants would recommend a loved one or friend to use this measure. Furthermore, we wondered whether these factors might affect the way they process the probability information conveyed. In contrast with previous research, perceived severity and the inconvenience caused by the preventive measure were assessed before exposure to the information in order to ensure that our participants’ responses were not influenced by the data provided.

In addition to previous beliefs, perceived severity and associated emotional reactions might also depend on the format in which numerical information is presented. In a previous study, we found that representing frequencies in the form of icon arrays makes them easier to understand than presenting them as Arabic digits, especially when having to infer posterior probabilities (Tubau et al., 2018).^1^ However, Petrova et al. (2015) concluded that visual aids only helped people for whom the medical information provided was not too affectively imbued; in contrast, people seeing the disease as extremely unpleasant or severe did not pay attention to the statistical information provided, and made their decision based on their previous beliefs of the effectiveness of screening or their fear of the disease. Also in the context of medical scenarios but with a different approach, Timmermans et al. (2008) found that human icons had more affective impact than frequencies or percentages, and risks presented as icons were judged as more likely. Nevertheless, format affected the decision in just one out of their four scenarios and some uncontrolled features of the scenarios make it difficult to extract general conclusions on the relationship between affective response and the intention to recommend preventive measures. All in all though, previous evidences suggested that presentation format was a variable to take into account.

Finally, it is worth noting that the effects of previous beliefs and affect might be also modulated by individual level of numeracy. Numeracy is defined as “the ability to process basic probability and numerical concepts” (Peters et al., 2006, p. 407). People with low numeracy are not only less accurate in estimating probabilities than their high in numeracy peers, but also more prone to frame, text complexity and numerical format effects (e.g., Peters et al., 2006, 2011; Johnson and Tubau, 2013). Furthermore, previous studies have found differences between people with low and high numeracy in both risk perception and commitment to take certain decisions, with people with low numeracy being less able to integrate probabilities and outcome information, particularly in affect-rich contexts (e.g., Pachur and Galesic, 2012). In contrast, emotions of people with high numeracy vary more in proportion to the probability of the loss than their peers (Petrova et al., 2014). Given these previous data, we decided to assess the numeracy of our participants by asking them to answer a selected sample of the items in the numeracy scale by Lipkus et al. (2001; see section Materials and Procedure).

Participants in our experiment were presented with two medical scenarios, one concerning breast cancer and the other regarding hypertension. These two medical conditions were selected because they were expected to differ in their perceived severity. Our sample consisted of university students, mainly women, in their early twenties: we hypothesized that, even though hypertension is more prevalent than breast cancer and has well-known possible negative consequences (e.g., a higher likelihood of suffering an ictus or heart attack), it would not be considered as lethal *per se*, especially by the sample in question. In order to verify our hypothesis and assess our participants’ previous beliefs, we explored how severe they considered the two medical conditions to be, before presenting them with any prevalence data. We also asked them about their beliefs regarding the two preventive measures they would have to recommend.

Subsequently, the two medical scenarios were proposed. Both included information on the prevalence of the disease, as well as data on a preventive measure. Health care campaigns often stress the positive effects of preventive measures and tend to omit the bothersome or even negative consequences of their use such as overdiagnosis. As a result, people may be well disposed to use them, even without considering the information provided (Petrova et al., 2016). So, in order to avoid an indiscriminate “yes” response to the recommendation, both health scenarios included also a drawback of it.

After the presentation of the medical scenario, we asked our participants again about the affect (worry) that the current information had aroused in them. We also wanted to determine how difficult they found it to understand the information provided. Finally, we asked them to rate the prior and posterior probabilities (see method) and to decide whether they would recommend this remediation measure. Our predictions were as follows. We expected that, prior to testing, participants would view breast cancer as more severe than hypertension. As for the perceived inconvenience of preventive measures, we did not have any preconceptions: we merely wanted to measure participants’ previous beliefs and feelings. Regarding the subsequent items, we expected that information on the more severe disease would also be considered as more worrying. We also predicted that, although participants would take into account the likelihood of the events, the weight of numerical information on the decision process might depend on the scenario: we expected that higher levels of worry and severity would make participants more likely to recommend preventive measures above and beyond the perceived probabilities.

**FIGURE 1:**
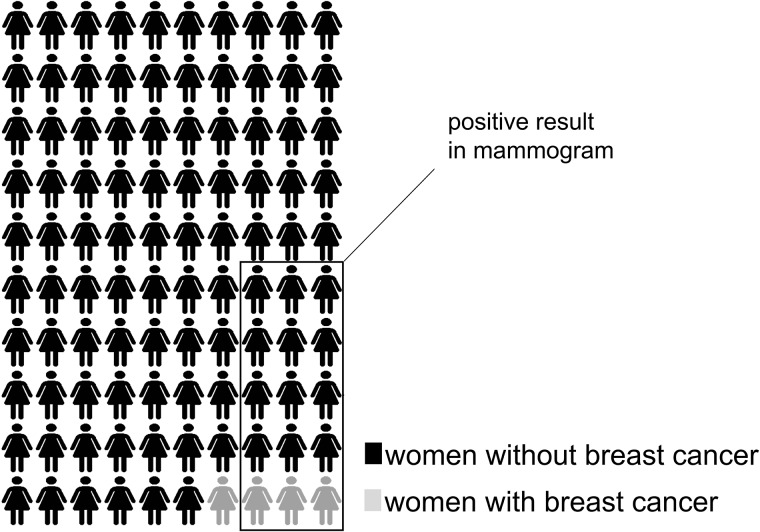
Iconic representation of the breast cancer problem (original version in Spanish and in color).

The statistical data for each scenario were presented either verbally, with quantities reported as natural frequencies in Arabic numerals, or through arrays of 100 icons (see **Figure 1**). Regarding the format, we aimed to test two alternative hypotheses. On the one hand, according to Timmermans et al. (2008), higher vividness of the risks displayed as icons should cause more affective response in participants than digits; this should increase the perceived probability and the commitment to recommend the preventive measure, especially in more severe medical situations. We considered that this effect might be maximized by the use of anthropomorphic figures, so we used restroom-like icons. On the other hand, based on the above mentioned benefit of icons for risk comprehension, we expected more sensitivity to the probability information for the ratings in this format.

Regarding the effect of numeracy, we hypothesized that people with low numeracy would consider information to be harder to understand than their high in numeracy peers, but they might also see it as more worrying and more likely to occur. This, in turn, might translate into a higher intention to recommend the preventive measure, especially in the more severe medical condition. In contrast, high-skilled participants might adjust their recommendation more to the probability ratings.

In sum, two scenarios differing in severity were used to investigate whether previous beliefs and affect related to a given medical condition and a possible preventive measure might influence the extent to which participants would recommend to use this measure. Given previous evidences of the relevance of these two variables in probability processing and decision making, format was manipulated and numeracy of participants was measured.

## Materials and Methods

### Participants

One hundred and forty-three Psychology students [115 women and 28 men, mean age = 23.37 (*SD* = 5.98)] from the University of Barcelona took part in this experiment as part of their course. Sensitivity analysis conducted with GPower (Faul et al., 2007) shows that for our main variable of interest, i.e., severity of the medical scenario, this sample size implies a minimal detectable effect of *f* = 0.15, which is considered to be small according to Cohen (1992).

Probabilistic reasoning and the Bayes rule were introduced only after this session. Participants were free to join in the experiment, and provided written consent for the use of their data for research purposes. They were debriefed in a subsequent session.

### Materials and Procedure

Participants were presented with two health scenarios concerning breast cancer and hypertension. Each scenario ended with the possibility of using a preventive measure (mammogram/low-sodium diet). Information was presented through icon arrays (see **Figure 1**) to 71 and in the form of natural frequencies (e.g., “3 of the 4 women with breast cancer and 12 of the 96 women without breast cancer receive a positive mammogram”) to the rest (*N* = 72). Participants were randomly assigned to each format condition. They were tested collectively, although each one had their own computer and solved the task individually. There were no time limitations for answering, although it took all participants between 15 and 20 min to complete the whole task.

The procedure was as follows^2^. An initial screen informed participants they would receive some data concerning the prevalence of breast cancer, and asked them to rate how severe they considered this disease and how inconvenient they thought mammograms were. Participants were required to respond using a 1-to-8 scale, on which a score of 1 meant hardly severe/inconvenient at all and 8 highly severe/inconvenient. A second screen reported the prevalence of breast cancer in women over 50, and the reliability of mammograms for its detection (prevalence: 4 of 100; hit rate: 3 of 4; false alarm rate: 12 of 96). It also noted that a positive mammogram result (true or false) might require a needle biopsy to confirm it. In order to avoid effects due to differences in individual memory, these data remained on the screen until participants had answered all the items on this medical scenario. Questions on this screen asked participants how difficult they had found it to understand the probability of suffering from breast cancer (1 = very easy; 8 = very difficult) and how worrisome (1 = not worrisome at all; 8 = very worrisome) they found the information provided. A third screen asked participants to think about a friend or relative in this age group and to rate the probability that she might suffer from breast cancer (prior probability; 1 = very unlikely; 8 = very likely), or that she might suffer from breast cancer if she had received a positive result in the mammogram (posterior probability). Finally, participants were requested to rate the extent to which they would recommend the mammogram to their friend or relative (1 = definitely not; 8 = definitely).

In the hypertension scenario, participants first had to rate the severity of the condition and the inconvenience of following a low-sodium diet. After this, they were provided with the base rate of women over 40 suffering from this medical condition as well as the rate of women following a sodium-rich diet with or without hypertension (prevalence: 20 of 100; hit rate: 12 of 20; false alarm rate: 24 of 80). They were also reminded that doctors often recommend a low-sodium diet, even though many people consider it to be unpleasant. Much as in the previous scenario, participants were required to rate the difficulty of understanding the information provided and how worrying it was (screen 2); the probability that a friend or relative of this age might suffer from hypertension, and the same probability if she followed a sodium-rich diet (screen 3). Finally, participants were told that their friend or relative was considering following a low-sodium diet and they were asked to decide whether they would recommend it.

We also assessed participants’ numeracy using the four items (see **Appendix**) rated by Peters et al. (2006) as the most difficult ones on the numeracy scale of Lipkus et al. (2001). Three of these items were the ones previously used by Schwartz et al. (1997). Participants answered these questions at the end of the session.

## Results

We had hypothesized that the two scenarios would differ in the previous beliefs and affect aroused by the medical condition and remediation presented and that this might have consequences in the likelihood of recommendations. Hence, our first analysis was devoted to confirm the existence of differences between the two medical scenarios. Given that we predicted that format and numeracy might also have an effect on the comprehension of the data and the affect aroused by them, we also entered these two variables into the analysis. Nevertheless, for the sake of comprehension, we will report the data concerning them separately.

We conducted an ANOVA for each dependent variable (responses to each question) with the medical scenario (breast cancer and hypertension) as a within-participant variable, and format (icons and natural frequencies) and numeracy (low and high) as between-participant factors. As for numeracy, participants were classified into two groups according to their performance on the numeracy questionnaire: they were considered as showing low numeracy (LN) if they had correctly answered two items or fewer (*N* = 69) and as having high numeracy (HN) if they had correctly answered three or four (*N* = 74).

### Effects of Scenario

For the sake of readability, *F* values, significance and descriptive statistics are presented in **Table 1**. As expected, the effect of scenario was significant for *perceived severity, worry, prior* and *posterior probability* and *recommendation rates*. For worry and probability ratings, scenario also interacted with format and numeracy (see below). More specifically, participants judged breast cancer to be significantly more severe than hypertension. The breast cancer scenario also raised more worry than the hypertension scenario, and the mammogram was recommended significantly more frequently than the low-sodium diet (see **Table 1**). Even if it was not theoretically relevant, finding no effects of scenario in difficulty helped us discarding that differences between scenarios were due to problems in comprehending one of them. As for probabilities, the fact that the ratings of the prior and posterior probabilities differed across scenarios, with higher ratings for hypertension, indicates that participants’ answers were sensitive to the disparity in the numerical information provided in each of them.

**Table 1:** Effects of the medical condition (severity of the scenario).

	Breast Cancer	Hypertension			
					
	Mean *(SD)*	Mean *(SD)*	*F* values	*p*	$\eta_{p}^{2}$
Perceived severity	7.20 (0.96)	5.89 (1.20)	*F*(1,139) = 160.40	<0.001	0.53
Inconvenience measures	4.17 (2.13)	4.09 (2.13)	*F*(1,139) < 1		
Difficulty	3.56 (1.87)	3.27 (1.64)	*F*(1,139) = 3.17	0.07	0.022
Worry	5.14 (1.68)	4.83 (1.60)	*F*(1,139) = 5.17	0.024	0.036
Prior probability	2.64 (1.48)	3.50 (1.51)	*F*(1,139) = 43.52	<0.001	0.23
Posterior probability	4.14 (1.89)	5.03 (1.71)	*F*(1,139) = 27.88	<0.001	0.16
Recommendation	7.34 (1.06)	5.60 (1.83)	*F*(1,139) = 117.95	<0.001	0.45


*Descriptive statistics [Mean and Standard Deviation (SD)] and comparison of responses to the breast cancer and hypertension medical scenarios.*

### Effects of Format

As above, here we report only significant effects. See **Table 2** for a detailed list of the descriptive statistics as well as *F* and *p* values.

**Table 2:** Effects of format.

	Breast Cancer		Hypertension				
					
	Icons	Frequencies	Icons	Frequencies			
							
	Mean *(SD)*	Mean *(SD)*	Mean *(SD)*	Mean *(SD)*	*F* values	*p*	$\eta_{p}^{2}$
Perceived severity	7.21 (.89)	7.19 (0.94)	5.74 (1.10)	6.04 (1.28)			
Inconvenience	4.15 (2.24)	4.18 (2.04)	4.12 (2.22)	4.05 (2.05)			
Difficulty	3.04 (1.76)	4.07 (1.84)	2.89 (1.59)	3.65 (1.62)	Format: *F*(1,139) = 12.75	< 0.001	0.084
Worry	5.24 (1.66)	5.04 (1.71)	4.99 (1.61)	4.67 (1.58)	Scenario x Numeracy x Format: *F*(1,139) = 3.88	0.051	0.027
Prior probability	2.49 (1.39)	2.78 (1.56)	3.52 (1.47)	3.47 (1.56)			
Posterior probability	3.89 (1.79)	4.39 (1.96)	5.20 (1.75)	4.86 (1.67)	Scenario x Format: *F*(1,139) = 6.29	0.013	0.043
Recommendation	7.35 (1.10)	7.34 (1.02)	5.83 (1.82)	5.38 (1.83)			


*Descriptive statistics [Mean and Standard Deviation (SD)] of the responses to each question and summary of results of the ANOVA investigating the differences between icons and natural frequencies.*

Format affected *difficulty, worry* (in interaction with scenario and numeracy; see below), and *posterior probability* rates (in interaction with scenario). That is, the data presented through icons were always judged to be *easier* to understand than data presented through written frequencies. Furthermore, participants who received the information in iconic format were more sensitive to variation in probabilities than those who saw it as frequencies: only the *posterior probability ratings based on icons* were correctly identified as differing across the scenarios [*t*(70) = 5.73, *p* < 0.001 and *t*(71) = 1.86; *p* = 0.06 for scenarios presenting icons and natural frequencies, respectively; see **Table 2**].

### Effects of Numeracy

Numeracy showed significant effects for *difficulty, worry* (in interaction with scenario and format) and *prior probability* rates (see **Table 3**). As expected, people with low numeracy rated the information provided in both scenarios as *more difficult* to comprehend than those scoring high in numeracy. They also rated breast cancer as *more worrying* than people with high numeracy when the data were presented through icons (means of worry rates were 4.8 and 5.8 for HN and LN, respectively; *t*(69) = 2.63, *p* = 0.01), but not in the case of natural frequencies (mean of worry rate in either group was 5; *t* < 1). Last, participants with low numeracy judged *breast cancer to be more likely* than their high in numeracy peers (see **Table 3**).

**Table 3:** Effects of numeracy.

	Breast Cancer		Hypertension				
					
	Low numerates	High numerates	Low numerates	High numerates			
							
	Mean *(SD)*	Mean *(SD)*	Mean *(SD)*	Mean *(SD)*	*F* values	*p*	$\eta_{p}^{2}$
Perceived severity	7.23 (0.91)	7.18 (0.92)	6.01 (1.26)	5.78 (1.13)			
Inconvenience	4.33 (2.25)	4.01 (2.02)	3.60 (1.99)	4.54 (2.17)	Scenario × Numeracy: *F*(1,139) = 7.07	0.009	0.048
Difficulty	4.03 (1.86)	3.12 (1.79)	3.80 (1.70)	2.78 (1.43)	Numeracy: *F*(1,139) = 14.65	<0.001	0.095
Worry	5.39 (1.69)	4.91 (1.65)	4.74 (1.74)	4.91 (1.46)	Scenario × Numeracy: *F*(1,139) = 4.43	0.037	0.031
					Scenario × Numeracy × Format: *F*(1,139) = 3.88	0.051	0.027
Prior probability	3.13 (1.74)	2.18 (1.01)	3.64 (1.57)	3.36 (1.45)	Numeracy: *F*(1,139) = 8.35	0.004	0.057
					Scenario × Numeracy: *F*(1,139) = 6.41	0.012	0.044
Posterior probability	4.32 (1.96)	3.97 (1.81)	5.25 (1.73)	4.82 (1.68)			
Recommendation	7.27 (1.13)	7.41 (0.99)	5.54 (1.81)	5.66 (1.86)			


*Descriptive statistics [Mean and Standard Deviation (SD)] of the responses to each question and summary of results of the ANOVA investigating the differences between low and high numerates.*

### Factors Influencing Recommendation

Our second analysis aimed at determining which variables might have affected decisions in a particular medical scenario. We first conducted a correlational analysis to check which variables (numeracy, format, scores on the items concerning disease severity, inconvenience caused by the preventive measure, difficulty to comprehend and worrying, as well as the estimated prior and posterior probabilities) significantly correlated with the likelihood to recommend the preventive measure. Subsequently we conducted a forced entry multiple regression for each scenario introducing the significant variables in the correlation analyses as potential predictors and using the scores in the recommendation item as dependent variable.

#### Scenario 1. Breast Cancer

Commitment to recommend correlated significantly with disease severity and worry (see **Table 4**). A model including these two variables accounted for 12% of the variance, *R*^2^ = 0.12, adjusted *R*^2^ = 0.11; *F*(2,140) = 10.17, *p* < 0.001. Disease severity and degree of worrying were both significant predictors of participants’ recommendation (β = 0.29, *p* < 0.001 and β = 0.17, *p* = 0.02, respectively) with disease severity receiving more weight.

**Table 4:** Correlation analysis for the breast cancer scenario.

	Severity	Inconvenience	Difficulty	Worry	Prior probability	Posterior probability
Severity						
Inconvenience	0.04					
Difficulty	0.003	-0.02				
Worry	0.08	0.08	0.13			
Prior probability	-0.09	0.01	0.35^∗∗^	0.27^∗∗^		
Posterior probability	-0.01	-0.03	0.31^∗∗^	0.14	0.29^∗∗^	
Recommendation	0.31^∗∗^	-0.05	0.06	0.20^∗^	0.11	0.08


**p < 0.01; *p < 0.05.

#### Scenario 2. Hypertension

Commitment to recommend correlated significantly with severity, worry, prior probability and posterior probability (see **Table 5**). A model comprising these variables reached significance, *F*(4,138) = 6.90, *p* < 0.001, and explained 16% of the variance in the recommendation of participants: *R^2^* = 0.16, adjusted *R*^2^ = 0.14. When looking at each particular predictor, only severity and posterior probability reached significance (β = 0.17, *p* = 0.02 and β = 0.25, *p* = 0.01, respectively) although worry closely approached it (β = 0.16, *p* = 0.054).

**Table 5:** Correlation analysis for the hypertension scenario.

	Severity	Inconvenience	Difficulty	Worry	Prior probability	Posterior probability
Severity						
Inconvenience	-0.02					
Difficulty	-0.08	-0.05				
Worry	0.23^∗∗^	0.05	-0.04			
Prior probability	0.09	-0.03	0.18^∗^	0.29^∗∗^		
Posterior probability	0.10	-0.08	0.15	0.36^∗∗^	0.64^∗∗^	
Recommendation	0.23^∗∗^	-0.13	0.02	0.29^∗∗^	0.20^∗^	0.31^∗∗^


**p < 0.01; *p < 0.05.

## Discussion

Ideally, making a decision implies considering the consequences of each choice as well as the probability that they may happen. However, when dealing with affect-rich situations such as deciding on medical treatments for ourselves or our loved ones, other factors seem to come into play. Our aim in this study was to investigate the role of previous beliefs and emotions in two medical situations differing in severity, i.e., in their negative consequences. Since the comprehension of probabilities is affected by the presentation format as well as by the numeracy skills of the recipient, these two variables were also controlled. A sample of university students were presented with two scenarios concerning breast cancer (the more severe disease) and hypertension (less severe) and two preventive measures that could be used to minimize their effects. Participants were required to complete a questionnaire regarding their beliefs, emotions and perception of the probabilities provided. Importantly, in order to ensure that *a priori* conceptions were measured, some of the items had to be answered before the presentation of the medical situation. The last question required participants to rate the extent to which they would recommend the preventive measures.

As expected, participants rated breast cancer as significantly more severe and worrying than hypertension and also recommended mammograms more frequently than low-sodium diets. Indeed, when analyzing the factors influencing recommendation, only worry and severity – not probability estimations – predicted the recommendation of mammograms. Therefore, it seems that when participants had to decide on the medical situation with the worse consequences (and presumably the more affectively charged) they completely ignored the likelihood data and based their decisions on previous beliefs and current emotions. This result corroborates previous findings indicating a “probability neglect” (Sunstein, 2002) in affect-rich choices, or the existence of an “affect heuristic” (Finucane et al., 2000).

It is worth mentioning that measures of severity and worry in the breast cancer condition did not correlate (see **Table 4**), which indicates that, although both items aimed to assess emotional reactions, they were based on different sources. Indeed, only the worry rating correlated significantly with the prior probability rating meaning that emotions measured after the presentation of the medical scenario might be more influenced by the perception of the actual data contained in it. This finding reinforces our idea that studies assessing the effects of *a priori* beliefs and emotions should measure them *before* the medical scenario is presented.

In contrast, the decision to recommend a low-sodium diet was best explained by taking into consideration the posterior probability, i.e. participants’ rating of the likelihood of suffering from hypertension provided a high-sodium diet was followed, and once again, severity. Therefore, our results seem to indicate that even though all medical problems are traditionally considered as affect-rich situations, probability information is not necessarily ignored; the psychological impact of probability information might depend on each particular medical condition, and more specifically, on how negatively it is perceived.

Before continuing, a specification must be made: the two scenarios differed in several aspects apart from severity, one of them being that a low-sodium diet can directly lower the chances of suffering from hypertension while mammogram only indirectly lowers the chances of having a breast cancer with worse consequences. Nevertheless, if this was the reason participants answered differently in the two scenarios, we would have expected that participants recommended the diet to a higher degree. In contrast, they were significantly more committed to recommend mammograms. The same would happen for prior probability: even if chances of suffering from hypertension are higher than those of having breast cancer, participants were more committed to recommend the preventive measure for cancer. Therefore, our results support the conclusion that differences across scenarios are due to the perceived severity of the disease described. If the medical condition is lived as severe and worrying, people do not look at likelihood or effectiveness: they simply recommend the proposed measure. In contrast, if the medical condition is not considered as severe (hypertension), they pay attention to the presented likelihoods, as shown by the significant correlation between posterior probability and willingness to recommend as well as by the fact that posterior probability was a significant predictor of the participant’s decision.

Our study also addressed two factors that are known to have an effect on the difficulty of processing and understanding likelihoods: format and numeracy. As far as format is concerned, there were two reasons for its manipulation in this experiment. On the one hand, most previous studies have found that presenting probabilities with visual aids, such as icon arrays, facilitates their comprehension compared to verbal formats such as frequencies or percentages (e.g., Brase, 2009; Garcia-Retamero and Hoffrage, 2013; Tubau et al., 2018). On the other hand, other studies have stressed that icons, being a more vivid representation of the likelihood of suffering bad consequences, may have a higher emotional impact in affect-rich contexts and may increase the perceived probability (e.g., Timmermans et al., 2008). Our results provided support for both positions. First, participants considered frequencies to be harder to understand than icon arrays. Moreover, when asked to rate the posterior probability of each medical scenario (20% vs. 33%) onto the 1-to-8 scale, they rated the probabilities displayed as icons differently but provided equivalent ratings for the scenarios presented as frequencies. We consider this as further evidence that probabilities represented iconically are processed in a more fine-grained way than frequencies and are easier to manipulate and translate into context-appropriate scales. As for the effects of format on emotions, our data also supported the hypothesis that icons have a higher emotional impact, although their effects were limited to specific circumstances: information provided in the form of icon arrays was judged as more worrying than that presented as frequencies only in the most severe scenario, and only by participants with low numeracy. Therefore, we confirmed that people with low numeracy are more affected by extraneous factors (i.e. factors that do not affect objective probabilities) than their peers (Reyna et al., 2009).

Numeracy had other effects as well. As expected, less skilled individuals judged the information provided as more difficult to understand. Moreover, they judged the likelihood of suffering from breast cancer to be higher than their high-skilled peers. According to Reyna et al. (2009), uncertainty about the meaning of numerical information might lead people with low numeracy to use other criteria, such as their affective interpretation of the situation to judge probabilities or make their decisions. Given that breast cancer was considered as the most severe situation, it might have been perceived by people with low numeracy as being more likely.

Overall, the results found in this research fit well inside the fuzzy trace theory proposed by Reyna (2008). According to this author, people extract *verbatim* and *gist* representations of the information conveyed. The former are literal, precise and quantitative representations, while *gist* representations answer the question “what does the information mean to that individual?,” a subjective interpretation of information that would be based on education, culture and experience, and would include the affective interpretation of this information. People prefer to operate on *gist* representations and therefore their actions might seem at odds with the objective information provided. Other approaches mentioned in the introduction also stress the role that previous beliefs, particularly emotions, play in the decision processes: the affect heuristic, the risk-as-feelings hypothesis, or the view of affect as a spotlight stress the fact that, when deciding in affect-rich contexts, outcomes are the main consideration and their actual likelihood would be either less important, dismissed (Finucane et al., 2000; Loewenstein et al., 2001; Pachur et al., 2014), or misunderstood (e.g., by the low numeracy participants in the present study).

Before concluding, we would like to mention two possible limitations of this study that future research might seek to overcome. First, despite the differences across scenarios and the effects of format and numeracy mentioned above, in general participants provided very high ratings of the probabilities in both medical scenarios and in both formats. This was probably due to the affect involved in these medical recommendations, but it may also have been due to the scale we used. Items had to be rated on an 8-point numerical rating scale with verbal anchors (e.g., hardly severe at all–very severe). A similar 7-point rating scale had already been used in the literature (see for instance Petrova et al., 2015 or Pighin et al., 2011). Furthermore, Haase et al. (2013) concluded that verbal rating, despite showing a slightly smaller correlation with the objective measures than frequency or percentage scales (0.91 vs. 1), was a sensitive measure and better predictor of intentions and decisions than other scales. However, the fact that only eight categories were used, and that the extreme ones were marked with verbal labels, might have led participants to provide a meaningful ordinal ranking of the probabilities (*gist*) displayed in the two scenarios instead of a precise (*verbatim*) estimation (Haase et al., 2013). Therefore, even if the 8-point probability ratings properly reflected the disparity of probabilities presented in both medical scenarios, they should not be taken as a direct extrapolation from a 0-100 scale.

Second, the decision participants had to make did not concern them but a friend or relative. Nevertheless, given that one of the scenarios talked about a medical condition that mostly affects women, we wondered whether they had felt particularly involved and reacted differently from men. Unfortunately, we had not controlled for gender and most of our participants were women (115 vs. 28 men). Therefore, the following information must be interpreted with caution; however, preliminary analyses suggest that there might be gender differences. Recommendation in the breast cancer scenario was best predicted by severity in the case of women, *R*^2^ = 0.14, adjusted *R*^2^ = 0.13; *F*(1,113) = 19.07, *p* < 0.001; β = 0.38, *p* < 0.001, and by posterior probability in men, although the data in this case failed to reach significance, perhaps because of the small sample *R*^2^ = 0.11, adjusted *R*^2^ = 0.07; *F*(1,26) = 3.24, *p* = 0.08; β = 0.33, *p* = 0.08. Gender effects, though, might not be due exclusively to the medical scenario; when we ran identical analyses on the hypertension situation, we found new differences between men and women. While the likelihood to recommend the preventive measure in women was best explained by worry, *R*^2^ = 0.06, adjusted *R*^2^ = 0.05; *F*(1,113) = 7.95, *p* = 0.006; β = 0.25, *p* = 0.006, men’s behavior was predicted by a model comprising posterior probability, β = 0.59, *p* = 0.001, severity β = 0.37, *p* = 0.018 and difficulty β = -0.31, *p* = 0.049, *F*(3,24) = 7.68, *p* = 0.001. Altogether, our current data seem to indicate that in rich-affect contexts women may pay less attention to numbers than men do, although better controlled future studies might want to confirm this point.

Summarizing, previous perception of the severity of a given medical condition modulates the use of probabilistic information for decision-making. Future efforts to ensure informed consent should not only focus on providing relevant data but may also require a reassessment of previous beliefs and emotions, and, if necessary, an attempt to correct them.

## Ethics Statement

This study was carried out in accordance with the recommendations of the University of Barcelona’s Bioethics Commission and the protocol was approved by this commission. All subjects gave written informed consent in accordance with the Declaration of Helsinki.

## Author Contributions

ÀC had the original idea and wrote the first draft of the manuscript. ÀC, JR-F, and ET participated in the design and reviewed the manuscript. JR-F created the questionnaire. ÀC and ET performed the statistical analysis.

## Conflict of Interest Statement

The authors declare that the research was conducted in the absence of any commercial or financial relationships that could be construed as a potential conflict of interest.

## Footnotes

1 Most of previous studies using icons presented them together with numerical frequencies and, perhaps because of this, mixed findings on format effects have been reported (see for example the meta-analysis by McDowell and Jacobs, 2017). Hence, to avoid potential confounds, our study used icons without the redundant numerical information.

2 For the sake of clarity, here we describe one particular session; however, the order of the two scenarios was counterbalanced across participants.

## Appendix

Items of the Numeracy Scale of Lipkus et al. (2001) used to test the participants’ numeracy:

(1) In the BIG BUCKS LOTTERY, the chances of winning a $10.00 prize are 1%. What is your best guess about how many people would win a $10.00 prize if 1,000 people each buy a single ticket from BIG BUCKS?
(2) Imagine that we roll a fair, six-sided die 1,000 times. Out of 1,000 rolls, how many times do you think the die would come up even (2, 4, or 6)?
(3) The chance of getting a viral infection is 0.0005. Out of 10,000 people, about how many of them are expected to get infected?
(4) In the ACME PUBLISHING SWEEPSTAKES, the chance of winning a car is 1 in 1,000. What percent of tickets of ACME PUBLISHING SWEEPSTAKES win a car?
